# An Immunothrombotic Extracellular Vesicle mRNA Profile Associated with Thrombosis in Lung Adenocarcinoma

**DOI:** 10.3390/ijms27125558

**Published:** 2026-06-19

**Authors:** María Marcos-Jubilar, Clara Fernandez-Arias, Carmen Herrero-Carrasco, Elizabeth Guruceaga, Karmele Valencia, Pablo Elizalde, Susana Inoges, Ramón Lecumberri, Josune Orbe

**Affiliations:** 1 Hematology and Cell Therapy Area, Clinica Universidad de Navarra, 31008 Pamplona, Spain; mmarcos.3@unav.es (M.M.-J.); clarafernaf@unav.es (C.F.-A.); cherreroc@unav.es (C.H.-C.); pelizaldeg@external.unav.es (P.E.); sinoges@unav.es (S.I.); 2 Instituto de Investigación Sanitaria de Navarra (IdiSNA), 31008 Pamplona, Spain; 3 Red de Investigación Cooperativa Orientada a Resultados en Salud (RICORS)-Cerebrovascular Diseases, Instituto Salud Carlos III, 28029 Madrid, Spain; 4 Bioinformatics Platform, CIMA-University of Navarra, 31008 Pamplona, Spain; eguruce@unav.es; 5 Program in Solid Tumors, Cancer Division, Cancer Center Clínica Universidad de Navarra (CCUN), CIMA-University of Navarra, 31008 Pamplona, Spain; kvalencia@unav.es

**Keywords:** lung adenocarcinoma, cancer-associated thrombosis (CAT), extracellular vesicles (EVs), transcriptomics, P-selectin (SELP), neutrophil degranulation, biomarkers

## Abstract

Venous thromboembolism (VTE) significantly impacts lung adenocarcinoma outcomes, yet current predictive tools lack precision. We investigated plasma extracellular vesicle (EV) mRNA as a liquid biopsy source to identify a pro-thrombotic molecular profile in VTE patients. Within a prospective cohort of 260 patients, we performed a retrospective nested case–control study, matching 10 VTE cases with 11 thrombosis-free controls. Plasma EV-RNA was analyzed via high-throughput sequencing. Differentially expressed genes (DEGs) were integrated with functional enrichment and explored across public non-cancer VTE datasets, buffy coat samples, and cell lines. RNA-seq identified 483 DEGs within the VTE patient EV compartment, predominantly linked to neutrophil degranulation (NETosis), inflammation, and coagulation. We identified a set of EV-associated candidate genes (SELP, ELANE, MYL9, DNASE1L3) distinguishing cancer-associated thrombosis from non-malignant VTE, along with transcripts (TFPI, FCGR2A) selectively enriched within the EV compartment relative to circulating blood cells. P-selectin (SELP) was the only significantly increased marker, providing the strongest complementary support at the protein level. This molecular state was detectable prior to the occurrence of VTE. Plasma EVs capture a multicellular mRNA profile, reflecting the systemic immunothrombotic activation in lung adenocarcinoma. Despite sample size limitations, these findings should be considered exploratory and hypothesis-generating, but they suggest the EV-derived mRNA in combination with circulating markers such as SELP may provide a framework for future studies aimed at improving risk stratification.

## 1. Introduction

Lung adenocarcinoma is characterized by a high incidence of venous thromboembolism (VTE), a complication that significantly worsens prognosis and complicates management [1,2,3]. Among the multifactorial pathogenesis of cancer-associated thrombosis (CAT), recent research has highlighted the pivotal role of extracellular vesicles (EVs) in mediating the hypercoagulable state observed in lung adenocarcinoma [4,5,6,7,8,9].

EVs, including exosomes, microvesicles, and apoptotic bodies, are membrane-bound particles released by tumor and host cells into the circulation. In lung adenocarcinoma, tumor-derived EVs are enriched in procoagulant molecules such as tissue factor (TF) and phosphatidylserine, which directly activate the coagulation cascade and promote thrombin generation [7,8]. Mechanistic studies have demonstrated that these EVs can also induce endothelial activation, disrupt the anticoagulant properties of the vascular endothelium, and enhance platelet aggregation, further amplifying the risk of thrombosis [5,7,8].

Notably, recent work has elucidated that large EVs from cancer cells can trigger neutrophil extracellular trap (NET) formation via delivery of specific molecular cargo (e.g., CYBA), leading to increased reactive oxygen species and chromatin decondensation, which in turn drive venous thrombosis [4]. Additionally, small EVs secreted by the pro-thrombotic lung niche have been shown to promote platelet aggregation [5]. Clinical studies confirm that circulating EV levels are higher in patients with lung adenocarcinoma and correlate with coagulopathy and thrombotic events [6].

Current risk models like the Khorana score often show suboptimal performance in lung adenocarcinoma, failing to reflect the tumor’s dynamic biological impact [10]. This highlights a critical unmet need for specific, mechanism-based biomarkers. Consequently, EVs have emerged as central mediators of the pro-thrombotic milieu, offering significant potential for improved risk stratification and the identification of novel therapeutic targets in this high-risk population [4,5,6,7,8].

Addressing this gap, we conducted a transcriptomic analysis of plasma-derived EVs to identify an exploratory candidate gene set associated with CAT in lung adenocarcinoma. Differentially expressed genes (DEGs) were identified in this discovery phase and subsequently evaluated in patient buffy coat samples, public non-cancer VTE datasets, and in vitro models. Furthermore, we evaluated the protein levels of these candidates in plasma to confirm their presence in circulation, providing a hypothesis-generating framework for future risk assessment.

## 2. Results

### 2.1. Demographic and Clinical Characteristics of the Selected Lung Patient Cohort

From a prospectively followed cohort of 260 patients with advanced lung cancer enrolled from 2014 to 2019, a total of 10 patients with thrombosis within 6 months following diagnosis and 11 patients matched for age, sex, diagnosis and stage without thrombosis were included in this study.

As shown in Table 1, patients’ mean age was 57.6 years, with 40% female patients. Age and sex distribution were similar in patients with/without thrombosis groups. Dyslipidemia and smoking were the most prevalent cardiovascular risk factors. Previous history of thrombosis was only present in patients with CAT and the median time from sampling to the onset of thrombosis was 1 month. Regarding cancer treatments, the most frequently used were chemotherapy and targeted therapy in both groups. The proportion of genomic alterations in EGFR or ALK was similar in both groups, and no alterations in ROS1 were detected.

### 2.2. Characterization of Extracellular Vesicles (EVs)

**Figure 1:**
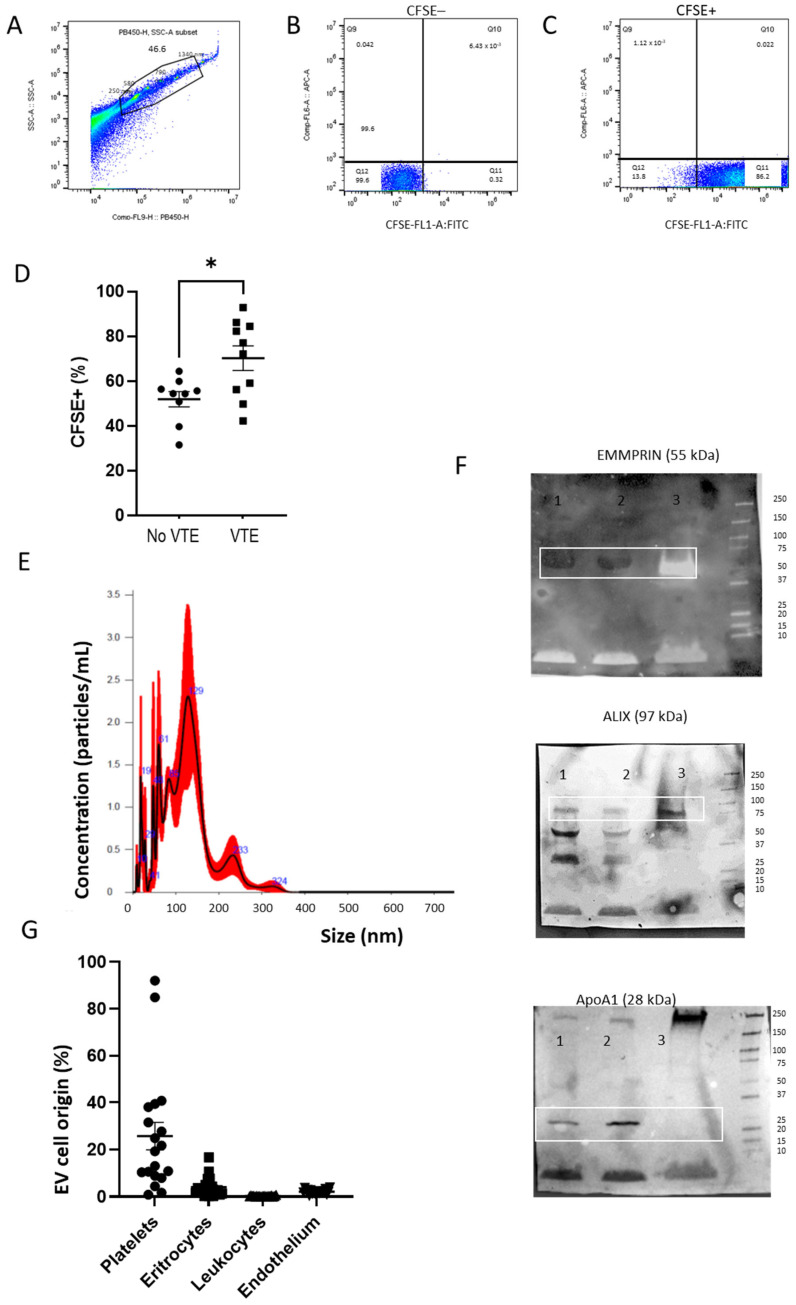
Characterization of plasma EVs from lung cancer patients: Flow cytometry plots for isolated EVs (**A**–**C**). EV working gate was defined using the violet side scatter (Violet-SSC) against the regular SSC using calibrated beads of sizes ranging from 250 nm to 1340 nm (**A**). Representative scatter plots of unstained (**B**) and CFSE-stained medium/large size EVs from plasma within the working gate (**C**). Scatter plot of CFSE-positive EVs according to thrombosis categories (**D**). Representative size distribution histogram obtained by NTA in plasma EVs of lung cancer patients (mean [SD]: 125.0 [±17.3] nm) and dot plot of thrombosis categories (**E**). Representative Western blot images of EV markers (Alix and EMMPRIN) and contaminants (ApoA1). Lane 1: EV lysate from patients with thrombosis; Lane 2: EV lysate from patients without thrombosis; Lane 3: Cell lysate from human aortic endothelial cells (HAEC). For the ALIX panel (middle), the primary target band is indicated at approximately 97 kDa. The additional lower molecular weight bands correspond to well-documented ALIX cleavage fragments, degradation products, or post-translational modifications typically observed in complex matrix (**F**). Quantification of plasma EV subpopulations by flow cytometry using specific antibodies against platelets (anti-CD41/61), leukocytes (anti-CD11b), endothelial cells (anti-CD62E) and erythrocytes (anti-CD235a). Data are presented as percentage of total events (n = 19) with SEM (**G**). CFSE: carboxyfluorescein N-succinimidyl ester; NTA: nanoparticle tracking analysis. Scatter plots show the mean and standard error mean (SEM). Statistical significance was determined using an unpaired *t*-test. * *p* < 0.05.

Prior to the transcriptomic experiments, laboratory analyses were performed to confirm the vesicular nature of the separated plasma EVs [11]. Flow cytometry analysis using calibrated beads to determine size showed that 62% of plasma EVs were able to uptake and process carboxyfluorescein N-succinimidyl ester (CFSE) (Figure 1A–C). In addition, CFSE-positive EVs were higher in the thrombosis group (Figure 1D), suggesting an increased shedding of membrane-intact vesicles specifically associated with the thrombotic state. By nanoparticle tracking analysis (NTA), plasma EVs showed a polydisperse distribution with particles with a mean size [SD] of 125 [±17.3] nm, and no significant differences in average size between patients with or without thrombosis. The mean concentration was 1.87 × 10^9^ ± 9.0 × 10^8^ particles/mL (Figure 1E), without statistically significant differences between patients with or without thrombosis. Western blotting analysis showed the presence of the EV markers Alix and EMMPRIN, as well as APOA1, a plasma lipoprotein marker, as the co-precipitated contaminant (Figure 1F). Finally, the cellular origin of plasma EVs, determined by flow cytometry, showed a predominance of platelet- and erythrocyte-derived EVs followed by EVs from endothelial cells and leukocytes (Figure 1G).

### 2.3. Transcriptomic Profiling of Plasma EVs Reveals an Exploratory Pro-Thrombotic Gene Set

**Figure 2:**
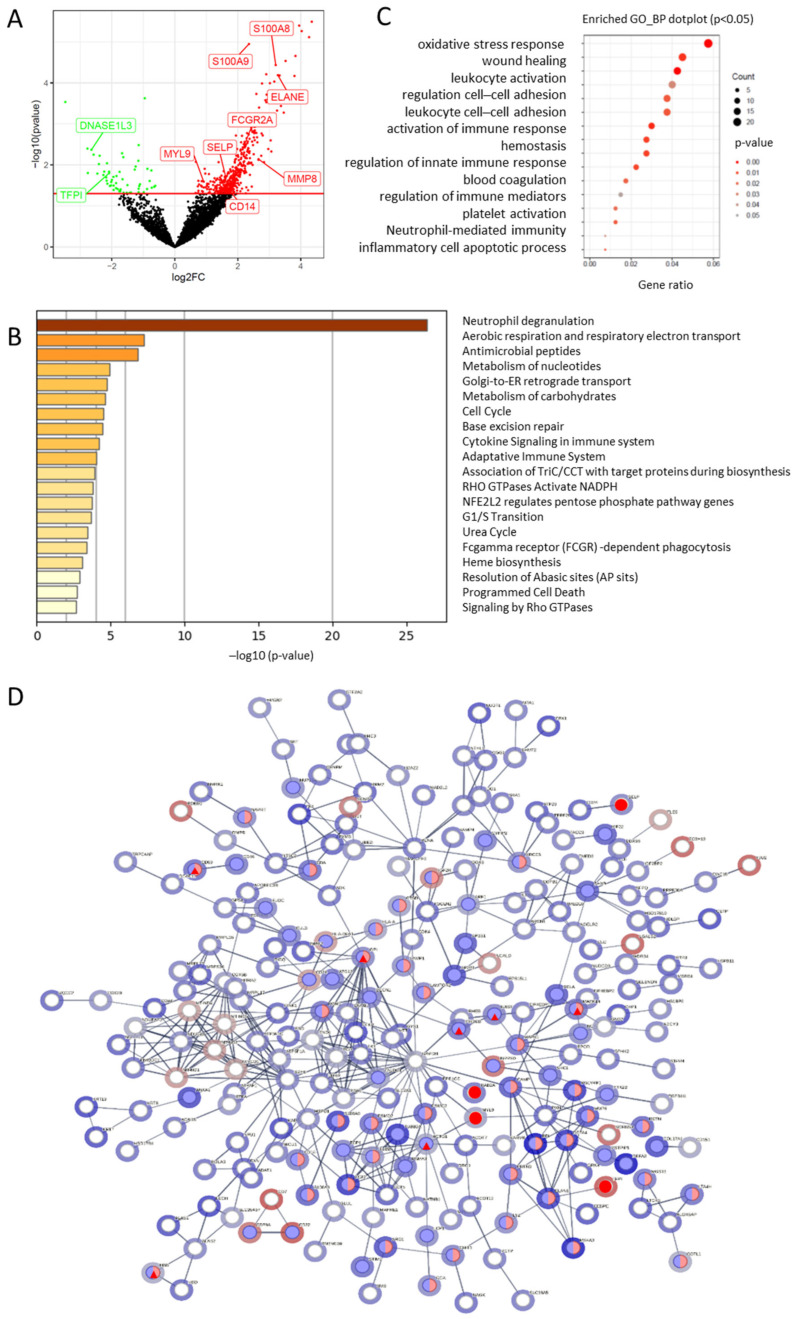
Bioinformatics analysis of plasma EVs of lung cancer patients after RNA-seq. Volcano plot showing significant DEGs between thrombosis-positive and -negative patients, highlighting the selected candidates (*p* < 0.05) (**A**). Top 10 Reactome terms from the functional enrichment analysis of DEGs between patients with and without thrombosis (**B**). The GO analysis for biological processes identifies enriched pathways between studied groups (**C**). Protein–protein interaction (PPI) networks with the DEGs between patients with and without thrombosis (*p* < 0.05) at the high confidence cutoff of 0.7 using STRING database (**D**). Blue and red colors on the edges indicate high and low levels, respectively. The blue, pink and red fillings represent the immune system, the neutrophil degranulation, and the blood coagulation pathways respectively.

Transcriptomic analysis of plasma-derived EVs detected 5081 genes across all samples, of which approximately 75% corresponded to protein-coding genes. Differential expression profiling identified a total of 483 DEGs between the two groups. Among them, 425 transcripts were upregulated and 58 were downregulated in patients who developed thrombotic events (Figure 2A). Consistent with the exploratory screening design of this phase, candidate identification was initially based on the nominal *p*-value threshold (*p* < 0.05), followed by biological prioritization to establish a broad basis for subsequent matrix validation. While the sequencing dataset included diverse RNA classes, downstream prioritization focused on protein-coding transcripts to directly align with the functional pathways under evaluation.

To refine the large number of differentially expressed genes, a biologically driven prioritization strategy was applied, combining differential expression, enrichment in biologically relevant pathways (including coagulation, inflammation, neutrophil activation, and NET formation), and network connectivity in protein–protein interaction (STRING) analysis. Enrichment analysis using the Reactome database (adjusted *p* < 0.05) showed significant overrepresentation of genes involved in key biological pathways, including inflammation, neutrophil degranulation, neutrophil extracellular traps (NETs), and oxidative metabolism (Figure 2B). These pathways were particularly enriched in the upregulated genes of the thrombosis group, suggesting a systemic activation of innate immune mechanisms that may contribute to thrombus formation.

Gene ontology (GO) analysis further confirmed enrichment in biological processes related to the response to oxidative stress, regulation of cell–cell adhesion, coagulation and platelet activation (Figure 2C).

STRING analysis of the DEGs identified protein–protein interaction networks enriched in genes involved in neutrophil degranulation (in pink), immune processes (in blue), and blood coagulation (in red) (Figure 2D).

Based on this integrative approach, candidate genes selected for downstream validation included ELANE, S100A8, S100A9, TFPI, F3 (TF), SELP, DNASE1L3, CD14, FCGR2A, MYL9, and MMP8. A summary of fold changes, adjusted *p*-values (FDR), and direction of change for the selected candidate genes is provided in Supplementary Table S1, while the complete differential expression results are available in Supplementary Table S2.

**Figure 3:**
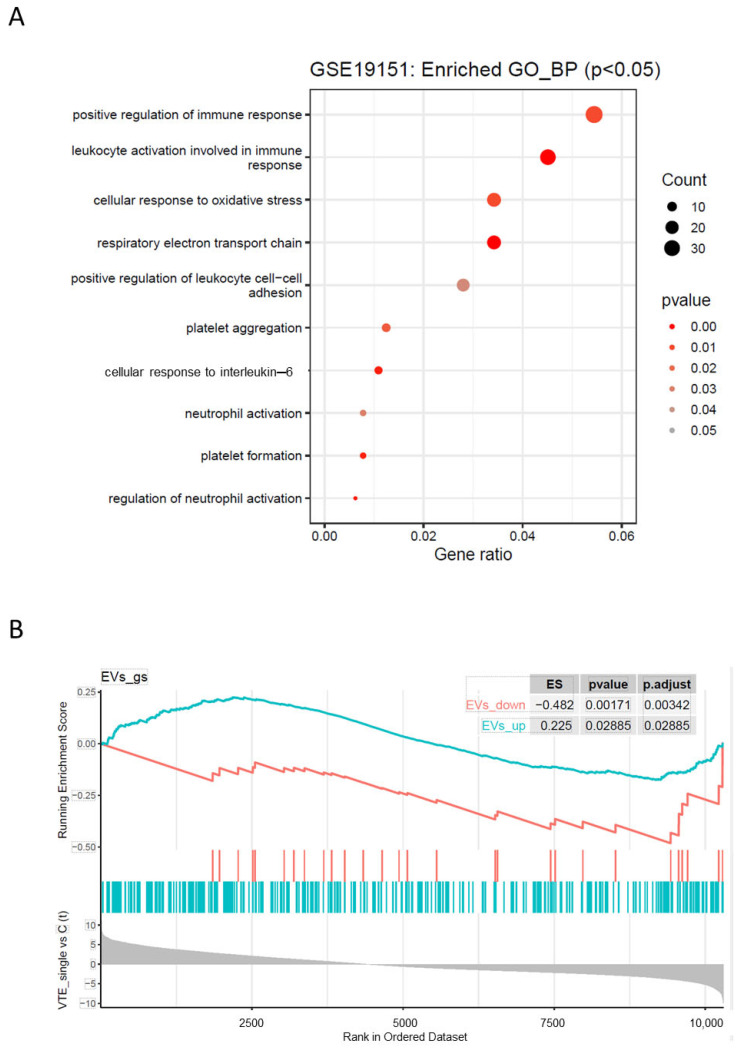
The transcriptional profile of DEGs in plasma EVs from patients with CAT overlapped with that of patients with VTE without cancer (GSE19151). (**A**) The GO analysis for biological processes identifies enriched pathways between patients with single VTE and healthy subjects. (**B**) Gene-set enrichment analysis (GSEA) of plasma EVs. The curve displays the running enrichment score (ES) of DEGs in EVs relative to the ranked list of genes in VTE patients according to up- or downregulation. Upregulated (green) and downregulated (red) gene sets include all differentially expressed genes identified in the analysis (425 upregulated and 58 downregulated genes, total 483 DEGs). The vertical lines in the middle of the figure mark the position of the corresponding genes in the ranked list. The enrichment score (ES) quantifies the enrichment of genes in up- and downregulated transcripts from plasma EVs relative to the ranked list of genes in patients with VTE ordered by lima t statistics (bottom).

Data from GSE19151 and plasma EVs were analyzed to understand whether the DEGs of plasma EVs from cancer patients with thrombosis overlapped with those from patients with VTE without cancer. The enriched GO biological pathways were similar to those shown in EVs, such as regulation of immune response, response to oxidative stress, regulation of cell–cell adhesion, neutrophil activation and platelet aggregation. Moreover, gene-set enrichment analysis (GSEA) showed a positive correlation between the expression profile of EV-derived transcripts from VTE cancer patients and a ranked gene list from whole-blood VTE datasets (Figure 3B).

Regarding selected genes, MMP8, S100A8, S100A9, and CD14 were differentially expressed in VTE patients both with and without cancer, suggesting a common inflammatory response to thrombosis. Conversely, the EV-mRNA candidate gene set (comprising SELP, ELANE, MYL9, and DNASE1L3), showed a more pronounced dysregulation in the lung cancer/VTE group, reflecting a specific systemic immune-thrombotic host response to malignancy. Notably, TFPI and FCGR2A exhibited preferential modulation within the EV compartment, highlighting a malignancy-specific molecular profile that remains masked in whole-blood analysis (Table 2).

### 2.4. Targeted Validation of Prioritized Candidates in Patient Buffy Coat and Plasma Samples

Considering that the mRNA content of EVs reflects the molecular status of the cells of origin, and that cells use EVs to deliver specific information to other target cells, we explored the selected genes in RNA extracted from the buffy coat. Using PrimeTime qPCR probe assays (with GAPDH as the reference gene), our goal was to determine the similarities and differences in the molecular mechanisms of thrombosis between the circulating EVs and their parent cells.

**Figure 4:**
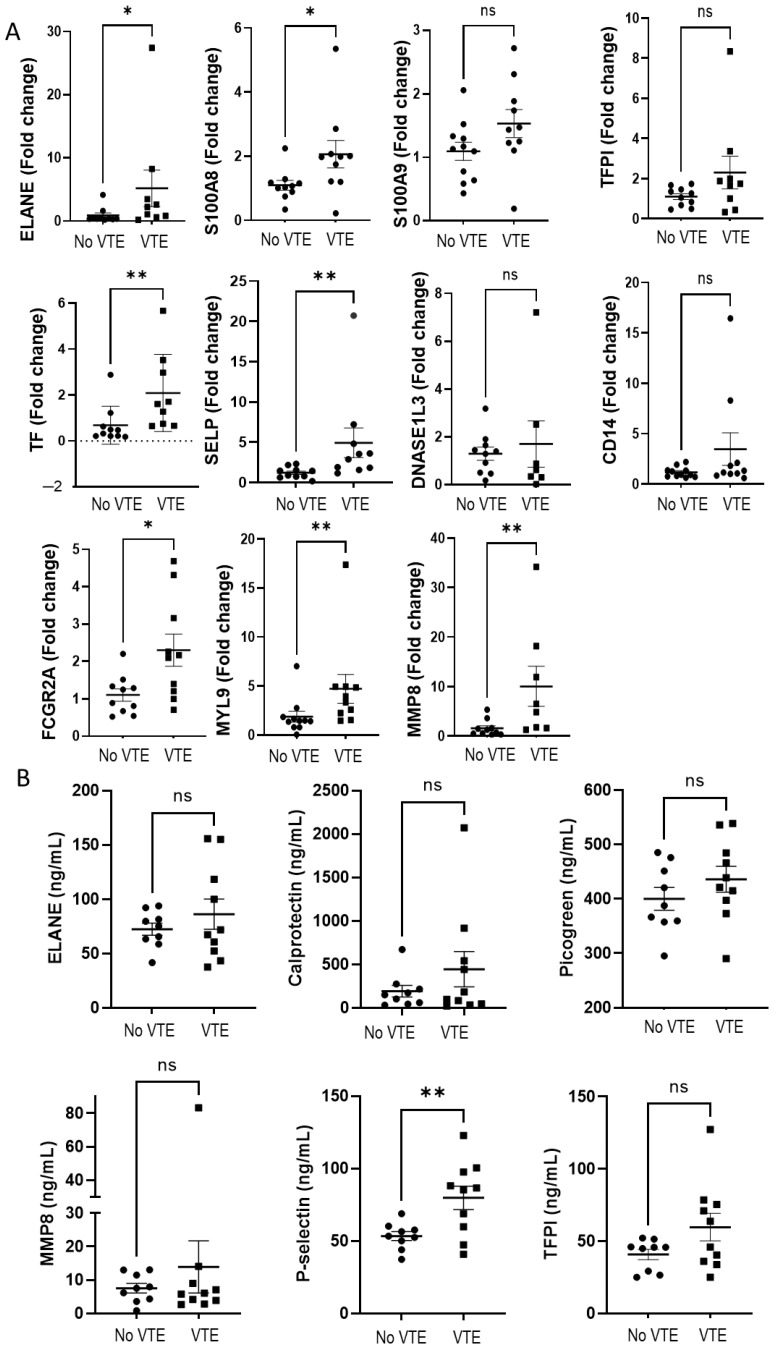
Gene expression and circulating protein levels of selected candidates in buffy coat (**A**) and plasma (**B**) samples from patients with and without VTE. mRNA levels are presented as fold change and circulating protein levels in ng/mL. Scatter plots show the mean and standard error mean (SEM). Statistical significance was determined using a Mann–Whitney test. * *p* < 0.05, ** *p* < 0.01, and ns = not significant.

While gene expression levels varied across samples, several candidates showed higher relative expression in CAT patients, such as MYL9, SELP, S100A8, MMP8, FCGR2A, ELANE, and F3 or TF (Figure 4A).

### 2.5. Circulating Protein Levels of Thrombosis-Related Markers in Plasma

Based on the initial findings of differential gene expression via RT-qPCR in the buffy coat, we proceeded to quantify the related protein levels in the plasma using ELISA. We measured ELANE, Calprotectin (S100A8/A9), PicoGreen, MMP8, P-Selectin, and TFPI. Among the analyzed proteins, P-selectin was the only marker showing a statistically significant increase in patients who developed thrombosis compared with non-thrombotic patients (Figure 4B).

To integrate transcriptomic, cellular, and plasma-level findings, a summary of the candidate genes and their corresponding biological relevance is provided in Table 3.

### 2.6. mRNA Expression of Differentially Expressed Genes in EVs from Lung Cancer Cell Cultures

**Figure 5:**
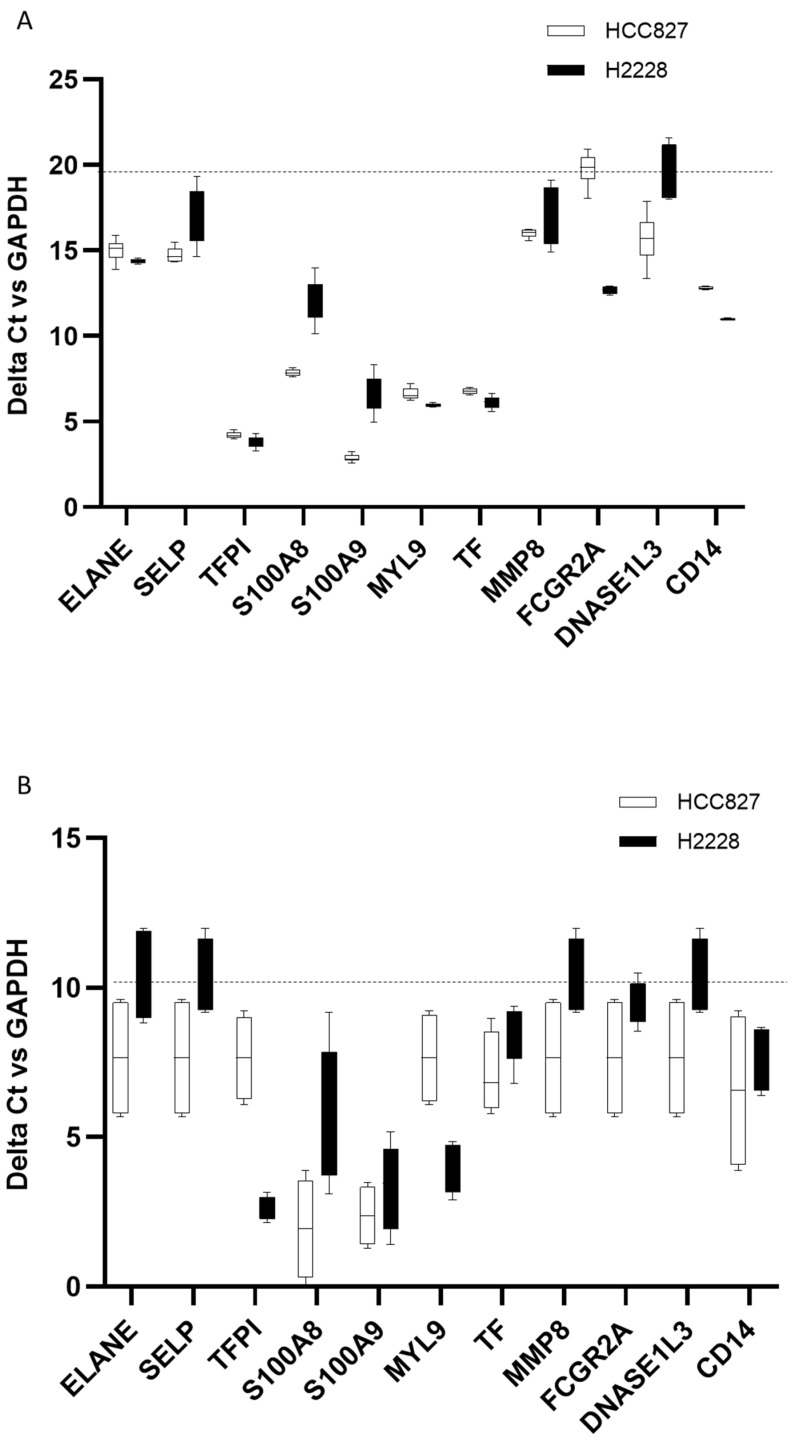
Relative gene expression (ΔCt) of candidate genes normalized to GAPDH. The dashed line indicates a ΔCt value of Ctgene = 40, representing the threshold for detection/non-expression. Gene expression data obtained from (**A**) cells, and (**B**) EVs isolated from HC827 and H2228 lung adenocarcinoma cell cultures are presented. Scatter plots show median with 95% CI.

Quantitative PCR analysis of selected genes established a baseline expression profile, where only six genes (TFPI, S100A8, S100A9, MYL9, F3 or TF, and CD14) were actively transcribed and subsequently packaged into their derived EVs. Importantly, the remaining five immune-associated genes (ELANE, SELP, MMP8, FCGR2A, and DNASE1L3) were scarce or almost absent in the tumor cell cultures. Similarly, these genes were undetected in cell culture EVs. These results suggest that while the six expressed genes are actively released by lung adenocarcinoma cells, the full thrombosis-associated EV profile observed in patients is influenced by non-tumor cells (e.g., platelets, neutrophils, monocytes) within the systemic circulation (Figure 5).

## 3. Discussion

Transcriptomic profiling of plasma EVs in advanced lung adenocarcinoma reveals 483 DEGs primarily involved in immune regulation, neutrophil degranulation, and coagulation signaling. An exploratory EV-associated set of candidate genes (SELP, ELANE, MYL9, and DNASE1L3) distinguished CAT from non-malignant VTE, suggesting a molecular profile linked to the neutrophil–platelet–endothelial axis. The consistent upregulation of pro-thrombotic and neutrophil-related transcripts in both plasma EVs and buffy coat cells indicates that the EV cargo reflects a broader, systemic immune–thrombotic activation, underscoring the exploratory nature and potential of these transcripts as candidate biomarkers. Supporting these findings, P-selectin was also significantly increased at the plasma level, reinforcing its established role as a circulating thrombosis risk biomarker in cancer.

In this context, the present study focused on the messenger RNA (mRNA) compartment of EV cargo. While non-coding RNAs (ncRNAs) are established circulating biomarkers, mRNA provides direct insight into protein-coding transcripts linked to functional pathways driving thrombosis, such as neutrophil activation, platelet aggregation, and coagulation. This vesicular mRNA cargo thus offers a real-time reflection of parent cell transcriptional shifts during the malignancy-driven pro-thrombotic state.

However, when evaluated in vitro, lung adenocarcinoma cell models did not fully recapitulate this EV-associated gene pattern, suggesting that the plasma EV biosignal is not exclusively tumor-derived. Rather, it likely represents a complex, composite signature originating from multiple cell types, including tumor, platelet, immune, and endothelial compartments, that collectively contribute to the patient’s systemic hypercoagulable state.

In lung adenocarcinoma, EVs act as potent drivers of CAT, simultaneously triggering platelet aggregation, initiating coagulation, promoting NETosis, and impairing endothelial anticoagulant functions [4,5,6,7,12,13]. Our results showed a predominance of platelet-derived EVs and a significant increase in CFSE+ EVs in patients with thrombosis, without a change in total EV concentration. These findings align with evidence demonstrating that the thrombogenic potential of EVs is primarily determined by their membrane composition and cargo rather than by total EV concentration alone [14,15,16,17], suggesting that the underlying differences between the groups might reflect biological changes in the EV content or cellular origin [18,19,20,21].

Bioinformatic analysis of EV-derived DEGs revealed a strong enrichment in pathways governing inflammation and coagulation, which is consistent with previous research showing that CAT is characterized by the upregulation of molecules mediating platelet aggregation, release of NETs, and coagulation/fibrinolysis in both tumor and host cells [7,12]. Prior studies in lung cancer patients with VTE have also shown enrichment in coagulation, inflammatory, and immune pathways, alongside specific genomic alterations (ALK, EGFR, and ROS1) [22,23]. Furthermore, EVs from lung cancer patients demonstrate increased procoagulant activity, largely mediated by TF and procoagulant phospholipids [7,15,24,25]. While predicting VTE risk solely from EV profiles remains limited, these findings support the hypothesis that EV-associated molecular cargo may represent candidate biomarkers and therapeutic targets for VTE, pending further validation.

Notably, the transcriptional profile of plasma EVs from our CAT cohort correlated with a public whole-blood dataset from non-cancer VTE patients (GSE19151), suggesting shared molecular pathways across thrombotic settings [26,27,28,29]. However, this comparison has important limitations. EV-derived RNA reflects a selectively packaged compartment, whereas whole-blood transcriptomics captures the global cellular RNA pool. Additionally, differences in analytical platforms (RNA-seq vs. microarray) and disease context limit direct comparability. Therefore, this analysis should be interpreted cautiously and considered supportive rather than as a formal external validation.

Interestingly, our comparative analysis between plasma EVs and circulating white blood cells (buffy coat) revealed distinct regulatory patterns. While genes like ELANE, S100A8, TF and SELP were upregulated in both compartments, suggesting activation of immune and vascular cells, specific transcripts including S100A9, TFPI, DNASE1L3, and CD14 exhibited preferential modulation and selective enrichment within the plasma EV compartment. This preferential enrichment supports the concept that EVs may capture molecular signals not fully represented in circulating cells, potentially reflecting contributions from endothelial or tumor compartments. Supporting this multi-level activation, P-selectin protein levels were also increased in plasma samples, aligning with the upregulation of SELP mRNA observed within the plasma EV. This concordance supports the biological relevance of the EV transcriptomic findings. Given that elevated circulating P-selectin is an established biomarker for thrombosis risk in cancer patients [30,31,32,33], this combined signal suggests that EV-derived mRNA and protein markers may provide complementary information for future biomarker development. EV-associated mRNA may reflect upstream transcriptional activation of immunothrombotic pathways, whereas circulating proteins such as P-selectin represent downstream functional markers of platelet and endothelial activation. An integrated overview of these multi-layer findings, including gene expression across EVs and buffy coat and corresponding plasma-level measurements, is summarized in Table 3.

However, these findings should be interpreted as exploratory, and the clinical utility of EV-derived biomarkers remains to be established. Future studies should evaluate whether the integration of EV-associated molecular signatures into existing clinical risk models, such as the Khorana or Vienna CATS scores, improves risk stratification in patients with cancer.

Beyond risk stratification, therapeutic targeting of the pathways identified in this study may represent a potential strategy. Preclinical models have shown that disrupting platelet–leukocyte interactions via P-selectin inhibition, or targeting NET formation using DNase I and PAD4 inhibitors can reduce thrombus burden without impairing physiological hemostasis or increasing hemorrhagic complications [34,35,36,37]. These findings are consistent with the multifactorial pathogenesis of CAT, where tumor-derived procoagulant signal is amplified by host immune and vascular responses [12,38].

This study has several limitations. First, the small cohort size (10 VTE cases and 11 controls) limits statistical power and generalizability, and therefore the findings should be considered exploratory. Second, the retrospective nested case–control design allows identification of associations but does not support causal inference, and prospective validation in larger, independent cohorts is required. Third, the low number of events limits statistical power and may increase variability in transcriptomic signals, affecting the robustness of the identified gene expression patterns. In addition, a proportion of patients with VTE had a prior history of thrombosis, which may reflect an underlying predisposition independent of cancer-associated mechanisms and may act as a potential confounding factor in the interpretation of the results. Notably, none of these patients were receiving anticoagulation at the time of sampling, suggesting that prior events were not recent; however, a baseline pro-thrombotic tendency cannot be excluded. Given the limited sample size, exclusion of these patients would have further reduced statistical power and potentially introduced additional bias. Therefore, these cases were retained and prior thrombotic history was considered a potential confounder. Furthermore, no functional assays were performed to directly evaluate the pro-thrombotic activity of extracellular vesicles or the biological effects of the identified transcripts, which limits mechanistic interpretation. Additionally, because bulk plasma EVs were analyzed, the exact cellular origin of the transcripts remains inferred and future studies using cell-specific EV isolation are needed. From a statistical perspective, the use of a nominal *p*-value threshold in the discovery phase increases the risk of false-positive findings, which was addressed through biological prioritization and multi-layer validation. Finally, the detection of ApoA1 indicates potential co-isolation of circulating lipoproteins, a known limitation of plasma EV isolation, and should be considered when interpreting EV-associated molecular profiles.

In conclusion, the integration of global EV transcriptomics with comparative circulating cell analysis allows for the exploratory identification of a distinct pro-thrombotic gene set. This profile emphasizes the role of neutrophil degranulation and endothelial/platelet activation as drivers of hypercoagulability in the oncological setting. These findings require validation in larger, independent prospective cohorts to confirm their reproducibility and generalizability before clinical application.

## 4. Materials and Methods

### 4.1. Patients

A nested case–control study was conducted within a cohort of 260 patients from the Clínica Universidad de Navarra, diagnosed with locally advanced or metastatic lung adenocarcinoma between 2014 and 2019. While clinical data and biobanked samples from this longitudinal cohort were analyzed retrospectively, the source cohort was followed prospectively for clinical events. This study was divided into an initial discovery phase based on RNA-seq of plasma EVs, followed by a multi-layered validation strategy encompassing patient buffy coat samples, external in silico datasets, plasma protein quantifications, and in vitro models. EDTA plasma (double centrifuged), buffy coat, and clinical data were provided by the Biobank of the Universidad de Navarra. All samples were processed following standard operating procedures, with the approval from the Human Ethics Committees and Scientific Review Boards (IP 2024.200), and in accordance with the Declaration of Helsinki and its subsequent amendments.

Within this cohort, 22 venous thrombotic events were recorded. For our nested molecular analysis, we strictly selected cases that developed thrombosis within the first six months after diagnosis. We prioritized patients with available plasma sample collected prior to the clinical onset of the thrombotic event and excluded individuals undergoing anticoagulation at the time of sampling. We ultimately selected n = 10 VTE cases and matched them by age, sex, diagnosis, and cancer stage with n = 11 controls from the same cohort who remained thrombosis-free during a one-year follow-up.

### 4.2. Characterization of EVs

#### 4.2.1. Nanoparticle Tracking Analysis (NTA)

Plasma EV size distribution and concentration were assessed in triplicate by NTA according to the manufacturer’s instructions (NanoSight NS300, Malvern Instruments Limited, Malvern, UK).

#### 4.2.2. Nanoscale Flow Cytometry

Carboxyfluorescein N-succinimidyl ester (CFSE, Sigma-Aldrich, Madrid, Spain) staining and its uptake were evaluated in plasma EVs by nanoscale flow cytometry. A total of 10 μL of isolated EVs from selected patients was labeled with 0.2 mM CFSE for 30 min at 37 °C and washed with 450 μL of wash buffer (2% BSA, 10 mM HEPES, 0.9% NaCl and 7.4 pH) for 70 min at 20,000× *g* (Mikro 22R, Hettich Zentrifugen, Tuttlingen, Germany). The supernatant was carefully removed, and the pellet diluted in 200 μL of PBS (filtered 1 × 0.22 µm, followed by 1 × 0.1 µm) and subsequently analyzed on the DxFlex cytometer (Beckman Coulter, Madrid, Spain) and the FlowJo software (10.8.1, BD Bioscience). To define the analysis gate for EVs, we compared the violet side scatter (Violet-SSC) to the standard SSC using calibrated polystyrene beads of 0.29, 0.58, 0.79, and 1.32 μm (NPPS-4K, Spherotech, IL, USA).

#### 4.2.3. Western Blot

A pool of 150 µL of EVs from three EV samples (50 µL per sample) from patients with thrombosis and patients without thrombosis was concentrated and lysed by thermal shock (3 × 37 °C-liquid N2). Samples from concentrated EVs and 20 µg of an endothelial cell lysate from human aortic endothelial cells (HAEC) were loaded in SDS-PAGE gel (4–20% Mini-PROTEAN TGX Stain-Free, Bio-Rad, Hercules, CA, USA) and transferred onto nitrocellulose membranes (Trans-Blot Turbo, Bio-Rad, Hercules, CA, USA). Blots underwent an overnight incubation with primary antibodies: 0.125 μg/mL Alix (mouse Anti-AIP1, clone 49/AIP1, #611620, BD Bioscience, Madrid, Spain), 1 μg/mL EMMPRIN (mouse monoclonal antihuman EMMPRIN/CD147, clone IT10C5, #21451471, Immunotools, Friesoythe, Germany), and 2 μg/mL ApoA1 (mouse anti human Apolipoprotein A1, sc-376818, Santa Cruz, Heidelberg, Germany). Peroxidase-conjugated secondary antibodies were applied as needed. Peroxidase activity was detected with a chemiluminescent substrate (TMA-6, Lumigen, Southfield, MI, USA) and images acquired with a ChemiDoc imaging system (Bio-Rad, Hercules, CA, USA).

### 4.3. Flow Cytometry Assessment of the Cellular Origin of Plasma EVs

To determine the cellular origin, 10 µL of plasma from selected patients was labeled with specific antibodies: 1:1.5 dilution of PE anti-human CD146-E for endothelium (clone S-Endo 1, #5050-PE100T, BioCytex, Marselle, France), 7.5 µg/mL of APC anti-human CD41/61 for platelets (clone A2A9/6, #359808, Biolegend, San Diego, CA, USA), 1:25 dilution of PC7 anti-human CD11b (clone Bear1, A54822, Beckman Coulter) for leukocytes, and 1:333 dilution of FITC anti-human CD235a for erythrocytes (clone KC16, B49206, Beckman Coulter). Plasma samples were incubated with the antibodies for 20 min at RT in darkness. The samples were run in the DxFlex cytometer (Beckman Coulter), and the results were analyzed with FlowJo software (10.8.1, BD Bioscience). Unlabeled plasma samples were used as negative controls.

### 4.4. Transcriptomic Analysis of Plasma EVs: RNA-Seq Library Construction

RNA-seq was performed in EVs isolated from plasma as previously described [39]. Plasma EVs were resuspended in 100 μL of lysis/binding buffer (Invitrogen, Paisley, Scotland). RNA transcripts were captured with dynabeads oligo (dT) (Invitrogen) and reverse-transcribed. RNA samples underwent a linear amplification by in vitro transcription followed by a fragmentation into 250–350 bp. Partial Illumina adaptor sequences were used, and a second reverse transcription reaction was performed. Full Illumina adaptor sequences were successfully added and the libraries sequenced (NextSeq 2000, Illumina, San Diego, CA, USA).

### 4.5. Bioinformatic Analysis

The quality of the data was evaluated with FastQC software (v0.11.8) and processing of the reads was carried out using Trimmomatic (v0.38) [40]. The resulting reads were aligned with STAR using GRCh38 human assembly and Gencode v38 as the genome annotation reference [41]. Then, duplicated reads were removed applying the UMI-tools dedup function [42]. Finally, expression levels were calculated with featureCounts software (v1.6.0) [43]. The obtained gene expression data were normalized using the analysis pipeline available in LIMMA package (TMM normalization and logCPM calculation using the Limma voom method) [44]. After quality assessment and outlier detection with R/Bioconductor [45], a filtering process was performed. Genes with read counts lower than 6 in more than 50% of the samples were excluded.

LIMMA was used to identify genes with differential expression. For each gene, statistical significance was assessed using moderated t-statistics, and both nominal *p*-values and adjusted *p*-values (false discovery rate, FDR, Benjamini–Hochberg method) were calculated, together with B-statistics (log-odds of differential expression). The complete differential expression results are provided in Supplementary Table S2.

For exploratory purposes, an initial list of differentially expressed genes (DEGs) was defined using a nominal *p*-value threshold (*p* < 0.05), without imposing a strict FDR cutoff, in order to retain potentially relevant biological signals given the limited sample size and the low RNA input characteristic of plasma EV samples.

To ensure robustness, FDR-adjusted results and B-statistics were systematically evaluated and are reported in the corresponding tables. Downstream prioritization of candidate genes was based on an integrative approach combining differential expression, pathway enrichment analyses, and biological relevance to thrombosis. Further functional and clustering analyses and graphical representations were performed using R/Bioconductor (v4.1.0) [45], and clusterProfiler [46]. In addition, Reactome gene set pathway analysis of differentially expressed genes (DEG) was performed based on Metascape database v3.5.20240101 (https://metascape.org/gp/index.html#/main/step1, accessed on 23 January 2025). Protein–protein interaction (PPI) pairs of DEGs were obtained from the Search Tool for the Retrieval of Interacting Genes (STRING, http://string-db.org/, version 12.0, accessed on 23 January 2025). In our study, a PPI high confidence score (0.7) was considered the threshold of significance to construct the PPI network.

### 4.6. Venous Thromboembolism Dataset

To gain insights from available public databases, the gene expression profile of VTE dataset GSE19151 was acquired from the Gene Expression Omnibus database (GEO). This dataset contains raw data of the expression profile from whole-blood samples of individuals with venous thromboembolism (VTE, n = 70) and the healthy control group (n = 63). From them, patients with single VTE, Caucasian/White race, and older than 20 years were selected, resulting in 23 VTE and 26 controls. Affymetrix microarray Human Genome U133A-2 data analysis included background correction and normalization using the RMA algorithm (Robust Multichip Average) [47], and a filtering process was performed to eliminate low expression probesets. Applying a criterion of expression value greater than 32 in 50% of the samples for each experimental condition, 17,059 probesets were selected for statistical analysis. LIMMA (Ritchie et al., 2015) [44] was used to find out the probesets with significant differential expression. Functional analyses previously described for plasma EVs were also performed in this case and the enrichment of differentially expressed genes in EVs (*p* < 0.05) was assessed using GSEA [46].

### 4.7. RNA Isolation and Candidate Gene Expression by RT-qPCR in Buffy Coat

Total RNA was extracted from 300 µL of frozen buffy coat using the Maxwell RSC SimplyRNA tissue kit (AS1340, Promega, Madrid, Spain). RNA quality and concentration were assessed with Qubit RNA BR Assay Kit, 500 (Q10211, ThermoFisher, Madrid, Spain).

Complementary DNA (cDNA) was synthesized from 1 µg of total RNA using random primers (Agilent, Santa Clara, CA, USA) and Oligo-dT (Agilent), and the M-MLV Retrotranscriptase (28025-013, Invitrogen). Gene expression was quantified by RT-qPCR on a QuantStudio 5 system (Applied Biosystems, Maastricht, The Netherlands) using TaqMan Fast Advanced Master Mix (4444557, Applied Biosystems) and specific PrimeTime qPCR probe assays (Integrated DNA Tecnologies, Leuven, Belgium) for candidate genes: ELANE (Hs.PT.58.40016354.g), TFPI (Ha.PT.58.2981702), SELP (Hs:PT.58.39915066), S100A8 (Hs.PT.58.19654111.gs), S100A9 (Hs.PT.58,20989743), CD14 (Hs.PT.56a.3118607.g), FCGR2A (Hs.PT.58.41033023), DNASE1L3 (Hs.PT.58.4210308), MYL9 (Hs.PT.58.1534321), MMP8 (Hs.PT.58.38488789), and F3 (Hs.PT.58.40870644), and housekeeping genes GAPDH (Hs.PT.39a.22214836) and HRPT1 (Hs.PT.58v.456221572). Data were expressed as the relative gene expression (ΔCt) of candidate genes normalized to GAPDH for cell cultures and their EV samples. mRNA expression data of patients with thrombosis are presented as fold change relative to patients without thrombosis after normalization with GAPDH.

### 4.8. Analysis of Plasma Proteins by Elisa

The concentrations of Elastase (ab119553, Abcam, Cambridge, UK), Calprotectin (S100A8/S100A9, 439708, Biolegend), MMP8 (DMP800B, R&D Systems), P-Selectin (DPSE00, R&D Systems, Minneapolis, MN, USA), double-stranded DNA (dsDNA) (Quant-iT™ PicoGreen™, P7589, ThermoFisher) and TFPI (DTFP10, R&D Systems) in plasma EDTA samples were measured using enzyme-linked immunosorbent assay (ELISA) kits, following the instructions provided by each manufacturer.

### 4.9. Cell Cultures

Adenocarcinoma, non-small cell lung cancer (H2228, ATCC, Manassas, VA, USA) sensitive to ALK inhibitors, and a lung adenocarcinoma (HCC827, ATCC) with an acquired mutation in the EGFR tyrosine kinase domain were cultured in RPMI with glutamine (61870-036, Gibco, Thermofisher) supplemented with 10% fetal bovine serum (FBS, A5256801, Gibco), 1% penicillin and streptomycin (PS, 100 U/mL and 100 µg/mL, 15070-063, Sigma-Aldrich). Cells were maintained in a humidified environment at 37 °C with 5% CO2, and the medium was renewed every 2–3 days.

Then, confluent cells were washed with PBS and incubated overnight in RPMI for 24 h. Supernatants were collected by serial centrifugations (5 min at 300× *g*; 10 min at 2500× *g*; and 70 min at 20,000× *g*) for EV isolation, and resuspended in 100 µL of HEPES buffer (10 mM HEPES, 0.9% NaCl and 7.4 pH). The cells were also collected with 2 mL of homogenization buffer (Maxwell RSC simplyRNA tissue kit, Promega), and stored at −80 °C until RNA isolation.

### 4.10. RNA Isolation from Cell Cultures

In total, 100 µL of EV suspension was incubated with 3.5 ng/µL of Proteinase K (Ref P4850, Sigma Aldrich, St. Louis, MO, USA) for 10 min at 37 °C. The reaction was subsequently neutralized by adding 17.6 µM of Proteinase K inhibitor (Ref 539470, Sigma Aldrich, St. Louis, MO, USA) for 10 min at room temperature. Samples were treated with 1 µg/mL of RNAse A (Ref EN0531, Thermofisher Scientific) for 20 min at 37 °C, followed by the addition of 5 µL of RNAseOUT (Ref 10777019, Invitrogen, Thermofisher). Total RNA was then extracted using the ReliaPrep RNA Tissue Miniprep System (Z6111, Promega).

Then, 200 µL of the cell homogenate was used to extract RNA using the Maxwell RSC SimplyRNA tissue kit (AS1340, Promega), as described above.

### 4.11. Statistical Analysis

Continuous data are presented as the mean ± standard deviation or median (interquartile range, IQR). Categorical data are presented as a number (percentage). The normality of the data was assessed with Shapiro–Wilk and Kolmogorov–Smirnov tests. Differences between categorical data were analyzed by the Chi square test. Differences between two independent groups were determined with the unpaired Student’s *t*-test for parametric variables, or Mann–Whitney U test for non-parametric variables. Statistical analysis was performed by GraphPad Prism 8.3.0 (GraphPad Software, Inc., San Diego, CA, USA).

## Figures and Tables

**Table 1:** Demographic and clinical characteristics of the lung patient cohort according to the presence of thrombosis.

	All	Thrombosis	
		NO (n = 11)	YES (n = 10)
Age (years)	57.6 ± 10.8	57.2 ± 10.1	58.1 ± 12.0
Sex (male, %)	59.1	58.3	60.0
IMC (kg/m2)	24.3 ± 3.6	24.9 ± 3.2	23.5 ± 4.1
Hypertension (%)	36.8	54.5	12.5
Dyslipidemia (%)	66.7	66.7	66.7
Diabetes (%)	11.1	11.1	11.1
Smoking (%)	47.6	36.4	60.0
History of previous thrombosis (%)	14.3	0	30.0
Time from sampling to thrombosis (month) *	1 (4.7)	-	1 (4.7)
**Treatments (%)**			
Chemotherapy	52.4	63.6	40.0
Chemotherapy + immunotherapy	9.5	9.1	10.0
Immunotherapy	4.8	0	10.0
Targeted therapy	28.6	18.2	40.0
Immunotherapy + targeted therapy	4.8	9.1	0
**Genomic alterations (%)**			
EGFR	40	45.5	33.3
ALK	13.3	12.5	14.3
Others (KRAS, BRAF, PIK3CA, TP53, MYC)	24.1	24.2	24
**Khorana VTE risk score (%)**			
1	38.1	45.5	30.0
2	52.4	36.4	70.0
>3	9.5	18.2	0

Mean ± SD; * median (interquartile range, IQR).

**Table 2:** Transcriptomic analysis of selected candidates in blood samples from patients with VTE without cancer (GSE19151), and plasma EVs from patients with lung cancer and VTE.

	Blood VTE vs. no VTE in Patients Without Cancer			EV VTE vs. no VTE in Patients with Lung Cancer		
Name	log2FC	AveExpr	*p*-Value	log2FC	AveExpr	*p*-Value
MMP8	0.479	5.098	0.003	2.659	−0.053	0.007
S100A8	0.843	7.327	0.000	3.204	7.298	0.000
S100A9	0.179	14.473	0.014	2.354	10.793	0.000
CD14	−0.390	12.347	0.000	1.660	0.744	0.036
SELP	−0.171	8.660	0.247	1.617	4.557	0.020
ELANE	0.228	6.723	0.266	3.272	3.065	0.000
MYL9	−0.071	8.338	0.795	0.916	10.472	0.026
DNAse1L3	0.117	5.476	0.192	−2.646	1.522	0.004
TF/F3	−0.123	5.499	0.023	NA	NA	NA
TFPI	NA	NA	NA	−2.087	1.637	0.016
FCGR2A	NA	NA	NA	2.153	2.691	0.004

NA = not available; positive log2FC indicates higher expression in VTE patients.

**Table 3:** Summary of the selected candidate genes, including their biological function, expression patterns across EVs and buffy coat, and plasma marker levels. EV expression refers to RNA-seq differential expression. Buffy coat expression was assessed by RT-qPCR and is presented as significant or non-significant. Plasma markers include proteins measured by ELISA and circulating double-stranded DNA (dsDNA) quantified by PicoGreen.

		VTE vs. no VTE				
Gene	Biological Function	EV Expression	Buffy Coat Expression	Plasma Marker (ELISA/PicoGreen)	Presumed Cellular Source	Relevance to Thrombosis
ELANE	Neutrophil elastase; NET formation	Upregulated	Upregulated	Not significant	Neutrophils	NET formation and thrombus stabilization
S100A8	Inflammation; neutrophil activation	Upregulated	Upregulated	Not significant (calprotectin)	Neutrophils/monocytes	Inflammation and endothelial activation
S100A9	Inflammation; neutrophil activation	Upregulated	Not significant	Not significant (calprotectin)	Neutrophils/monocytes	Inflammation and endothelial activation
SELP	Platelet adhesion molecule (P-selectin)	Upregulated	Upregulated	Significant (increased)	Platelets/endothelium	Platelet activation and adhesion
MYL9	Cytoskeletal regulation and contractility	Upregulated	Upregulated	Not assessed	Platelets/smooth muscle	Platelet activation and vascular contraction
F3 (TF)	Initiator of coagulation cascade	_	Upregulated	Not assessed	Tumor cells/monocytes	Clot initiation
TFPI	Inhibitor of tissue factor pathway	Downregulated	Not significant	Not significant	Endothelium	Regulation of coagulation
DNASE1L3	NET degradation	Downregulated	Not significant	Not assessed	Immune cells	Regulation of NET turnover
CD14	Innate immune receptor	Upregulated	Not significant	Not assessed	Monocytes	Inflammatory signaling
FCGR2A	Immune receptor; platelet activation	Upregulated	Upregulated	Not assessed	Platelets/immune cells	Platelet-immune interactions
MMP8	Matrix remodeling enzyme	Upregulated	Upregulated	Not significant	Neutrophils	Vascular remodeling and inflammation
dsDNA	NET formation marker	_	_	Not significant	NET release (neutrophils)	NET burden and pro-thrombotic state

## Data Availability

Sequence data that support the findings of this study have been deposited in NCBI’s Gene Expression Omnibus [48] and are accessible through GEO Series accession number GSE313339 (https://www.ncbi.nlm.nih.gov/geo/query/acc.cgi?acc=GSE313339, accessed on 18 June 2026). All other relevant data supporting the findings of this article are available from the corresponding authors upon reasonable request.

## Supplementary Materials

The following supporting information can be downloaded at https://www.mdpi.com/article/10.3390/ijms27125558/s1.

## Abbreviations

The following abbreviations are used in this manuscript:

CAT	Cancer-associated thrombosis
CFSE	Carboxyfluorescein N-succinimidyl ester
DEGs	Differentially expressed genes
EV	Extracellular vesicle
GO	Gene ontology
GSEA	Gene-set enrichment analysis
HAEC	Human aortic endothelial cells
IQR	Interquartile range
NTA	Nanoparticle tracking analysis
NET	Neutrophil extracellular trap
PPI	Protein–protein interaction
STRING	Search Tool for the Retrieval of Interacting Genes
SEM	Standard error mean
TF	Tissue factor
VTE	Venous thromboembolism
