# Supplementary material for: An Immunothrombotic Extracellular Vesicle mRNA Profile Associated with Thrombosis in Lung Adenocarcinoma
Source: Int J Mol Sci. 2026 Jun 19;27(12):5558. doi: 10.3390/ijms27125558 (PMC13299458; doi:10.3390/ijms27125558)
Supplement: Supplementary file 1 [file ijms-27-05558-s001.zip › ijms-4355337 Supplementary Table S1.pdf]

**Supplementary Tables:**

Table S1. Differential expression analysis of selected candidate genes in plasma extracellular vesicles (EVs) from lung adenocarcinoma patients with and without venous thromboembolism (VTE), obtained using the limma voom pipeline. Data are presented as log2 fold change (log2FC), average expression (AveExpr, logCPM scale), moderated t-statistic (t), nominal p-value (P.Value), adjusted p-value (adj.P.Val, Benjamini–Hochberg false discovery rate), and B-statistic (log-odds of differential expression). Positive log2FC values indicate higher expression in VTE patients.

| gene_name | log2FC | AveExpr | t | P.Value | adj.P.Val | B |
|---|---|---|---|---|---|---|
| ELANE | 3.27 | 3.065 | 4.493 | 6.47E-05 | 0.029 | 1.674 |
| TFPI | -2.09 | 1.637 | -2.526 | 0.01585 | 0.415 | -3.066 |
| SELP | 1.62 | 4.557 | 2.432 | 0.01990 | 0.451 | -3.440 |
| S100A8 | 3.20 | 7.298 | 4.678 | 3.66E-05 | 0.023 | 2.144 |
| S100A9 | 2.35 | 10.793 | 5.060 | 1.12E-05 | 0.011 | 3.066 |
| DNASE1L3 | -2.65 | 1.522 | -3.043 | 0.00425 | 0.249 | -1.973 |
| CD14 | 1.66 | 0.744 | 2.173 | 0.03616 | 0.499 | -3.735 |
| FCGR2A | 2.15 | 2.691 | 3.049 | 0.00418 | 0.249 | -1.989 |
| MYL9 | 0.92 | 10.472 | 2.325 | 0.02558 | 0.472 | -4.227 |
| MMP8 | 2.66 | -0.053 | 2.840 | 0.00723 | 0.314 | -2.416 |

Table S2. Full. unfiltered output of the differential expression analysis performed on plasma extracellular vesicle (EV) RNA-seq data from lung adenocarcinoma patients with and without venous thromboembolism (VTE). The table includes all 5.081 expressed genes and the corresponding statistical metrics generated using the limma voom pipeline: log2 fold change (log2FC). average expression (AveExpr. logCPM). moderated t-statistic (t). nominal p-value (P.Value). adjusted p-value (adj.P.Val. Benjamini–Hochberg false discovery rate). and B-statistic (log-odds of differential expression). This dataset is provided to ensure full transparency of the analysis. (See excel file).
